# Acute Restraint Stress Induces Long‐Lasting Synaptic Enhancement by Inhibiting AMPK Activation in AD Model Mice

**DOI:** 10.1111/cns.70335

**Published:** 2025-03-18

**Authors:** Ming Wang, Baoyuan Jin, Jihoon Jo

**Affiliations:** ^1^ School of Public Health Health Science Center, Ningbo University Ningbo China; ^2^ The First Affiliated Hospital of Ningbo University Ningbo China; ^3^ Department of Biomedical Sciences Chonnam National University Medical School Gwangju South Korea

**Keywords:** Alzheimer's disease, AMPK, hippocampus, long term potentiation, stress

## Abstract

**Background:**

Alzheimer's disease (AD) is characterized by a gradual synaptic loss. The progression of AD severely affects late‐phase long‐term potentiation (L‐LTP), which is essential for long‐term memory consolidation.

**Aim:**

We have previously demonstrated the beneficial effects of acute restraint stress (ARS) on hippocampal LTP in AD mouse models. This study aimed to verify the effects and potential mechanisms of ARS on the maintenance of hippocampal L‐LTP in two AD mouse models.

**Materials and Methods:**

5xFAD and Tg2576 mice underwent a 30‐min body immobilization protocol to induce ARS, followed by electrophysiological recordings of L‐LTP (> 3 h) in the CA1 region of thehippocampus.

**Results:**

The ARS‐exposed group exhibited significantly enhanced L‐LTP compared to the control group. Maintenance of L‐LTP requires new protein synthesis and signaling via the mammalian target of rapamycin (mTOR) pathway. Our findings revealed that ARS increased hippocampal adenosine triphosphate (ATP) production and reduced AMPK activity. Inactivation of AMPK and subsequent activation of the mTOR pathway were strongly associated with the ARS‐facilitated enhancement of L‐LTP. Furthermore, our experiments using the mTOR inhibitor rapamycin demonstrated that it effectively prevented the enhancement of L‐LTP following ARS, underscoring the pivotal role of mTOR in this process.

**Conclusion:**

ARS may significantly modify AMPK activation and mTOR regulation in L‐LTP, potentially triggering the mechanisms of long‐term memory consolidation in AD mouse model mice. Identifying these underlying mechanisms could help promote the development of novel pharmaceutical agents for the treatment of AD.

## Graphical abstract

Acute restraint stress regulates ATP production in the hippocampus of Alzheimer's disease model mice and inhibits abnormal activation of AMPK under the pathologic condition, resulting in the removal of the suppression of the mTOR pathway, which is involved in the enhancement and maintenance of L‐LTP.

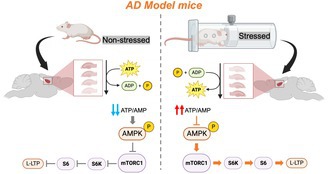

## 1 Introduction

LTP represents an increase in synaptic transmission that underlies the cellular process of memory formation [1]. Early‐phase LTP (E‐LTP) induction is rapid, and its expression does not require new protein synthesis, which is sustained 1 h after the delivery of high‐frequency stimulation (HFS). However, a persistent form of synaptic plasticity, L‐LTP, requires new protein synthesis, which occurs 2–3 h after an HFS protocol [1, 2, 3, 4]. The mammalian target of rapamycin (mTOR) kinase complex modulates protein translation through the phosphorylation of p70S6 kinase (p70S6K) and then subsequently phosphorylates the ribosomal protein S6 (rpS6) [5, 6, 7]. mTOR, a key modulator of L‐LTP, regulates the translational capacity by promoting protein synthesis [8, 9, 10]. Furthermore, rapamycin, a specific mTOR inhibitor, suppresses sustained hippocampal LTP [11]. Moreover, the knockout of upstream mTOR molecules leads to synaptic plasticity deficits and memory loss [12]. However, the potential role of upstream mTOR molecules in L‐LTP maintenance remains unclear.

Adenosine monophosphate (AMP)‐activated protein kinase (AMPK) is a central mediator of energy homeostasis in the central nervous system (CNS) [13, 14, 15, 16]. Generally, a reduction in cellular ATP levels regulates AMP, which then activates AMPK via phosphorylation at threonine 172 (Thr172), resulting in accelerated ATP production and reduced ATP consumption [17, 18, 19]. Acute stress can increase the release of ATP in the hippocampus [20, 21], indicating a potential effect of acute stress on AMPK activity. In the brain, AMPK hyper‐activation inhibits mTOR, resulting in impaired axonal growth [22]. The inhibition of AMPK activity directly enhances synaptic plasticity by mediating mTOR [23]. mTOR signaling is involved in several processes that regulate neurodevelopment and neuronal plasticity. Thus, dysregulation of mTOR signaling has been linked to several brain disorders associated with neuronal dysfunction, such as neurodegeneration and neurobehavioral alterations [24]. The AMPK activators significantly prevent the L‐LTP expression, whereas L‐LTP maintenance increases with AMPK inhibitors [25]. Furthermore, rapamycin completely diminishes the effect of AMPK inhibitors on L‐LTP enhancement [9]. This strongly suggests that the AMPK–mTOR pathway is involved in a protein synthesis dependent on LTP.

AD is an irreversible and progressive neurodegenerative disease that is associated with deficits in cognitive function and memory loss. The “amyloid cascade hypothesis” has provided the main theoretical construct for AD [26, 27]. The best understood AD pathogenesis in CNS is attributed to a loss of plasticity, representing a hippocampal LTP deficit [28, 29].

Stress affects how significant experiences are interpreted and serves as a benchmark for future events through memory [30]. The hippocampus is a target of stress events that cause atrophy of dendrites, and chronic stress events suppress the neurogenesis of hippocampal neurons [31]. However, it has been recently reported that both acute stress and stress hormones can enhance hippocampal LTP through dynamic molecular alterations under normal and abnormal conditions [32, 33, 34, 35]. Furthermore, we have previously demonstrated the effect of an ARS protocol on LTP facilitation in the hippocampus of AD model mice, which was mediated by glucocorticoids [36]. This indicates a potential role for acute stress events in impaired synaptic plasticity. However, further clarification is needed regarding the cellular and molecular mechanisms mediating the long‐lasting effects of ARS on hippocampal LTP enhancement in AD mouse models.

To address this question, we utilized the transgenic AD mouse models—5xFAD mice and Tg2576 mice. Field recording of hippocampal LTP was performed after body immobilization for 30 mins, and the molecular alterations were examined. We found that the effective duration of ARS facilitated LTP up to 3 h after the tetanus protocol, which is similar to our previous study. Importantly, L‐LTP induced by ARS within the AD mice hippocampus was accompanied by a strong inhibition of AMPK activity and an increase in mTOR downstream molecular expressions. In addition, a specific mTOR inhibitor fully abolished L‐LTP enhancement using the ARS protocol. These results establish a mechanism for AMPK‐mTOR signaling‐controlled protein synthesis in the acute stress‐facilitated L‐LTP. Owing to its potential role in learning and memory, the ability to restore the LTP deficit under pathological conditions may be beneficial for therapy.

## 2 Material and Methods

### 2.1 Animals

Male 5‐month‐old 5xFAD transgenic mice (APP KM670/671NL, APP I716V, APP V717I, PSEN1 M146L, PSEN1 L286V) were obtained from The Jackson Laboratory (Bar Harbor, ME). 9‐month‐old Tg2576 mice (APP KM670/671NL) were obtained from Taconic (Rensselaer, NY). The experiment was carried out in accordance with the recommendations of “96 Guidance for Animal Experiments,” established by the “Animal Ethics Committee” at Chonnam National University, and the protocol was approved by the ‘Animal Ethics Committee’ at Chonnam National University.

### 2.2 Acute Restraint Stress and Hippocampal Slices Preparation

5xFAD and Tg2576 mice were physically restrained in well‐ventilated 50 mL Falcon tubes for 30 min. Control mice were housed in their usual cages under normal conditions. Mice were sacrificed by cervical dislocation and then decapitated. The brains were quickly removed and submerged in ice‐cold artificial cerebrospinal fluid (aCSF) containing (in mM) 124 NaCl, 3 KCl, 26 NaHCO_3_, 1.25 NaH_2_PO_4_, 2 CaCl_2_, 1 MgSO_4_, and 10 glucose. The hippocampus was transversely sectioned (400‐μm thick) using a McIlwain tissue chopper (Mickle Laboratory Engineering Co. Ltd.) and stabilized for 1 h via perfusion with aCSF gassed with a 95% O_2_/5% CO_2_ mixture at room temperature.

### 2.3 Electrophysiological Recordings

Hippocampal slices were transferred to a recording chamber perfused with oxygenated aCSF (32°C–34°C). To record field EPSP, a stimulating bipolar electrode was placed along the Schaffer‐collateral pathway. Field EPSP was assessed with a glass microelectrode prepared on a micropipette puller (P‐1000; Sutter Instrument, Novato, CA, USA) with 3 M NaCl (3–5 MΩ) inside. After a stable baseline was established for 30 min, LTP was induced by two tetanus stimulation strains (100 Hz for 1 s with a 30 s‐interval). Field EPSP was assessed for 3 h after tetanus. Data were collected using an NI USB‐6251 data acquisition module (National Instruments, Texas, USA), amplified by an Axopatch 700B amplifier (Axon Instruments, CA, USA), and using WinLTP software (http://www.winltp.com).

### 2.4 Golgi‐Cox Staining and Spine Density Analysis

Golgi‐Cox staining was performed using a FD Rapid Golgi Stain Kit (FD NeuroTechnologies Inc., USA), following the vendor's protocol. Briefly, brains were isolated and fixed in 4% paraformaldehyde for 24 h (*n* = 3 per group from three animals). The brain tissue was then cut into 1.5–2 mm thick slices and rinsed in 1X PBS for 5 min. Subsequently, the slices were immersed in an impregnation solution (a mixture of equal volumes of solutions A and B, prepared 24 h prior to use) and stored at room temperature for 2 weeks in the dark. Finally, the tissue was transferred into solution C and maintained at 4°C for 3 days in the dark. Afterwards, it was sliced using a McIlwain tissue chopper (Mickle Laboratory Engineering Co. Ltd.) to a thickness of 100 μm, then mounted on gelatin‐coated microscope slides with a drop of solution C. After air‐drying naturally at room temperature overnight, the coronal sections were immersed in the developer solution (solution D: E: double distilled water = 1:1:2) for 10 min. The sections were then dehydrated through successive steps in alcohol at increasing concentrations (50%, 75%, 95%, and 100%) before being closed with slide cover slips. Images of dendrites within the hippocampus area were captured using the CaseViewer 2.3.0 system (3DHISTECH, Budapest, Hungary). Four neurons from the CA1 region (12 neurons in total per group) displaying dendritic trees without truncations and isolated from neighboring neurons were selected. Segments of dendrites ranging from 70 to 100 μm were selected from both basal and apical dendrites of each pyramidal neuron for the analysis. The number of spines and the total length were quantified using ImageJ (NIH, Bethesda, MD, USA). The spine number was divided by the length of dendritic segments to obtain the final spine density (spines/μm) from each sample.

### 2.5 Measurement of ATP Concentration

After hippocampal slice preparation, the slices were incubated in oxygenated aCSF at room temperature. Hippocampal slices were collected at 100, 200, and 300 min after ARS performance, respectively. Hippocampal ATP concentrations were evaluated by an ATP bioluminescence assay kit (11699695001, Sigma‐Aldrich). Hippocampal slices were homogenized with 12% perchloric acid (Sigma‐Aldrich) on ice and neutralized with 30% KOH to PH 7.8, followed by centrifugation at 10,000 *g* for 10 min. The supernatants were collected and incubated with luciferase reagent‐containing lysis buffer for 5 min. Standard curves were constructed by using known concentrations of ATP (provided with ATP bioluminescence assay kit). TD‐20/20 luminometer (2020‐000, Turner Designs, San Jose, CA, USA) was used for the measurement of the luminescent signal of each sample in triplicate. The level of ATP in different samples was obtained from standard curves. Furthermore, sample pellets were reconstituted with 1× PBS and used to determine the protein concentrations by using the Pierce BCA assay kit (Thermo Fisher Scientific, Waltham, MA, USA). Divide the ATP level by the total protein amount to obtain the final ATP concentration (pmol/mg protein) from each sample.

### 2.6 Western Blots of Hippocampus

Hippocampus was lysed in cold RIPA buffer (AKR‐190; Cell Biolabs, San Diego, CA, USA) with a protease inhibitor cocktail (210205; Cell Biolabs Inc.). Then, 30–40 μg of proteins were separated on 10%–12% SDS–polyacrylamide gel and transferred to PVDF membranes (Millipore, Bedford, MA, USA). The expressions of p‐AMPK (Thr172) (#2535), total‐AMPK, p‐rpS6 (Ser235/236) (#2211), rpS6, p‐p70S6K (Thr389) (#9205), p70S6K, or β‐Actin (Cell Signaling, Danvers, MA, USA) were detected by each antibody. The immunoblots were incubated with specific secondary antibodies (Abcam) for 2 h at room temperature, and the bands were obtained using the ECL detection system (Millipore, Bedford, MA, USA).

### 2.7 Immunofluorescence

Mouse brain slices (20 μm) were mounted on collagen‐coated glass slides (Thermo Scientific, Waltham, MA, USA), fixed in acetone solution for 10 min, washed in Tris‐buffered saline, and then exposed to methanol for 5 min. Nonspecific labeling was prevented by incubating the sections in 5% bovine serum albumin (Sigma‐Aldrich) for 1 h before incubating overnight at 4°C with the following specific primary antibodies (1:500 dilutions): p‐AMPK (Thr172) (Invitrogen, CA, USA) and primary antiserum to NeuN (1:1000). The slices were washed 3 times (5 min each) with phosphate‐buffered saline with 0.1% Tween 20 and then incubated for 2 h in the dark with AlexaFluor 488 goat anti‐rabbit IgG and AlexaFluor 594 goat anti‐mouse IgG (1:500; Invitrogen). All brain slices were counterstained with 1 μg/mL 4′,6‐diamidino‐2‐phenylindole (DAPI; Sigma‐Aldrich) and visualized with a confocal microscope (Carl Zeiss, Oberkochen, Germany).

### 2.8 Rapamycin

Rapamycin (Medchemexpress, USA) was dissolved in 100% ethanol, stored at −20°C, and freshly dissolved in an aqueous solution of 5% Tween 80 and 5% PEG 400 immediately before use. A single dose of rapamycin (40 mg/kg) was intraperitoneally injected for each mouse prior to acute restraint stress performance.

### 2.9 Data Analysis

Data are presented as mean ± standard error of the mean (S.E.M.). The Shapiro–Wilk test was used to determine data distribution. For comparison between two groups, an unpaired Student's *t*‐test was used. For comparison among three or more groups, a one‐way analysis of variance (ANOVA) was utilized by individual. Pearson's correlation coefficient was used to measure correlation. The value *p* < 0.05 was considered statistically significant.

## 3 Results

### 3.1 L‐LTP Is Enhanced in the Hippocampus of Stressed 5xFAD and Tg2576 Mice

**FIGURE 1:**
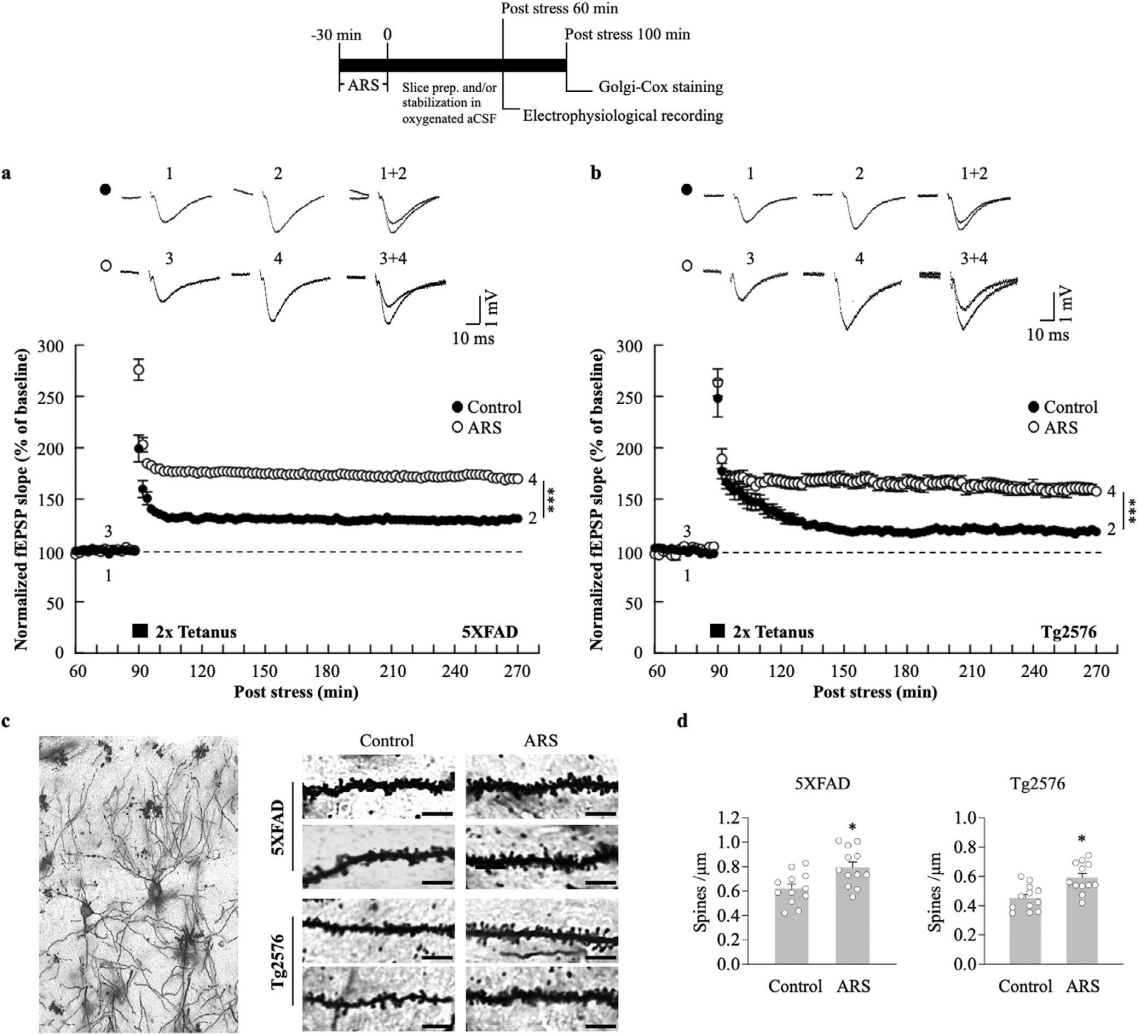
ARS facilitates hippocampal L‐LTP induction and maintenance in both 5xFAD and Tg2576 mice. (a) Facilitation of L‐LTP induction (HFS: 2 times tetanic stimulation at 100 Hz, 100 pulses; interstimulus interval, 30 s) in the CA1 region of 5xFAD mice hippocampal slices effected by ARS (open circle) compared with the control (closed circle), *n* = 6 per group from six animals. (b) Facilitation of L‐LTP induction in the CA1 region of Tg2576 mice hippocampal slices effected by ARS (open circle) compared with the control (closed circle), *n* = 6 per group from six animals. (c) Golgi‐Cox staining was used to detect the morphology of CA1 pyramidal neurons. Representative dendritic segments are reported for each group. (100× magnification, scale bar = 5 μm). (d) The plots represent the average density of spines calculated for each group. *n* = 12 dendritic segments from three animals per group. Data are expressed as means ± S.E.M. Differences were considered significant at *p < 0.05, ***p < 0.001. ARS, acute restraint stress; Control, non‐stressed; fEPSP, field excitatory postsynaptic potential.

To examine the hypothesis that ARS can increase the L‐LTP in the AD mouse hippocampus, we acutely prepared hippocampal slices from stressed and non‐stressed 5xFAD or Tg2576 mice. Field excitatory postsynaptic field potentials (fEPSPs) were recorded in the hippocampal CA1 region and LTP was induced using an HFS protocol (two trains of 100 Hz, 100 pulses). For data acquisition, L‐LTP was recorded for 180 min post‐HFS. ARS exerted a sustained effect on LTP maintenance; the fEPSP slopes of L‐LTP significantly increased in hippocampal slices from the stressed 5xFAD mice compared to the non‐stressed mice hippocampal slices (ARS: 169.8% ± 3.1% vs. control: 131.2% ± 1.7%, *p* < 0.01, *n* = 6, Figure 1a). The enhanced fEPSP slopes were also observed in the stressed Tg2576 mice hippocampus compared to the non‐stressed mice (ARS: 157.5% ± 4.3% vs. control: 118.4% ± 2.7%, *p* < 0.01, *n* = 6, Figure 1b). L‐LTP is a persistent form of synaptic plasticity that requires new protein synthesis, supporting the hypothesis that it is based on the formation of new synapses [37]. To evaluate whether ARS‐facilitated L‐LTP was also accompanied by structural changes in the hippocampus, we performed the Golgi‐Cox staining of the hippocampal CA1 pyramidal cells. The results showed an increase in dendritic spine density following ARS treatment in 5XFAD and Tg2576 mice compared to the non‐stressed mice (Figure 1c,d).

### 3.2 Acute Restraint Stress Prevents AMPK Activation in the Hippocampus

**FIGURE 2:**
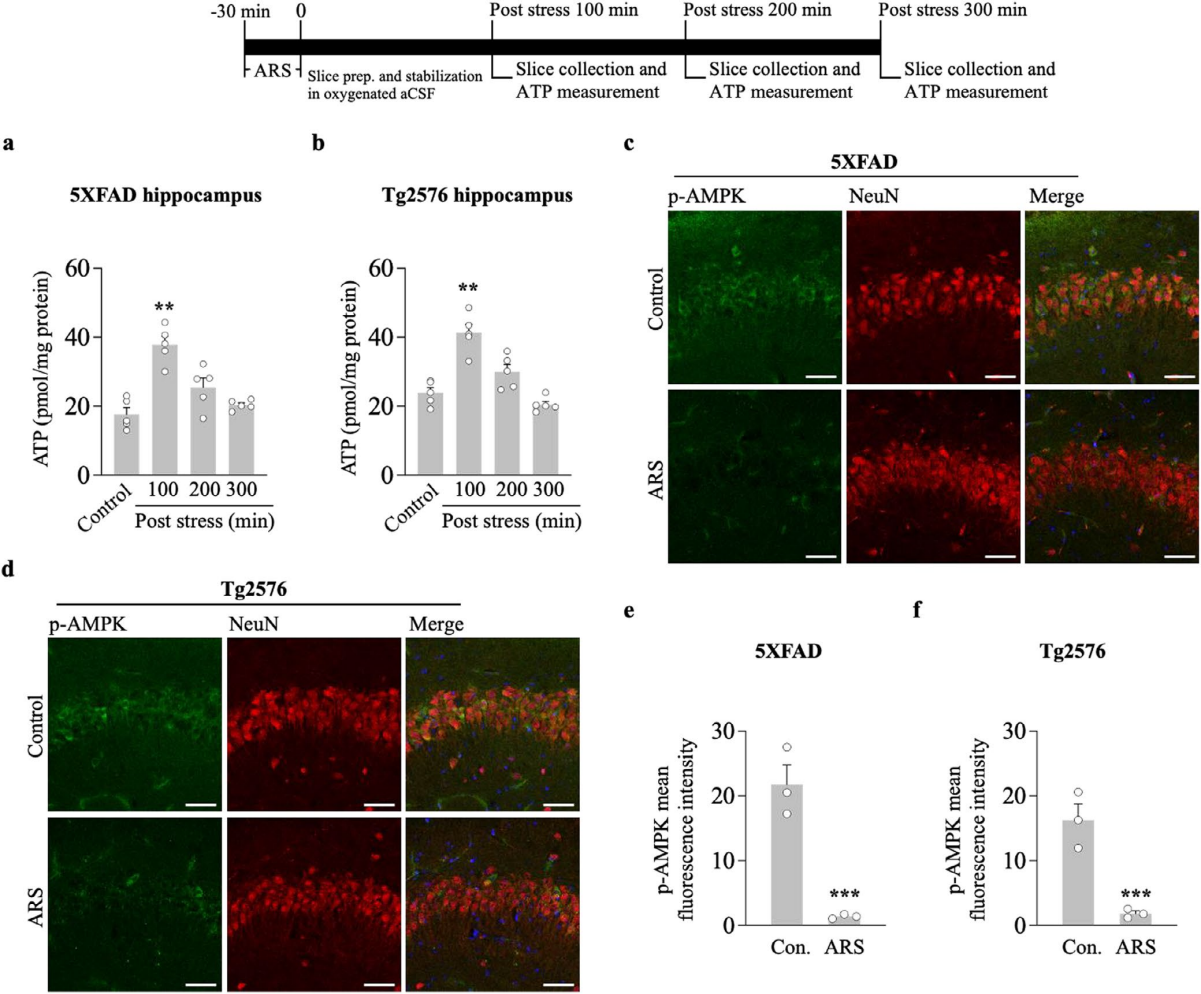
ARS increases ATP concentration and inhibits AMPK in 5xFAD and Tg2576 mouse hippocampus. 5xFAD mice (a) and Tg2576 mice (b) hippocampal ATP concentration was detected by ATP bioluminescence assay at different times after ARS, and then compare to the ATP concentration of control mice (*n* = 5 per group from five animals). (c, d) ARS inhibit AMPK in cell body of the pyramidal neuron at CA1 region of hippocampus from 5xFAD mice (*n* = 3 per group from three animals) and Tg2576 mice (*n* = 3 per group from three animals). Anti‐p‐AMPK immunoreactivity is displayed in green. NeuN is displayed in red. (e, f) The quantification of pAMPK immunofluorescence is presented as the mean‐ fluorescence intensity. Data are expressed as means ± S.E.M. Statistical analyses were used one‐way ANOVA with Turkey's multiple comparison post hoc test. For the comparison of two groups, statistical analyses were conducted using an unpaired Student's *t*‐test. Differences were considered significant at **p < 0.01, ***p < 0.001 versus control. Scale bar = 50 μm.

**FIGURE 3:**
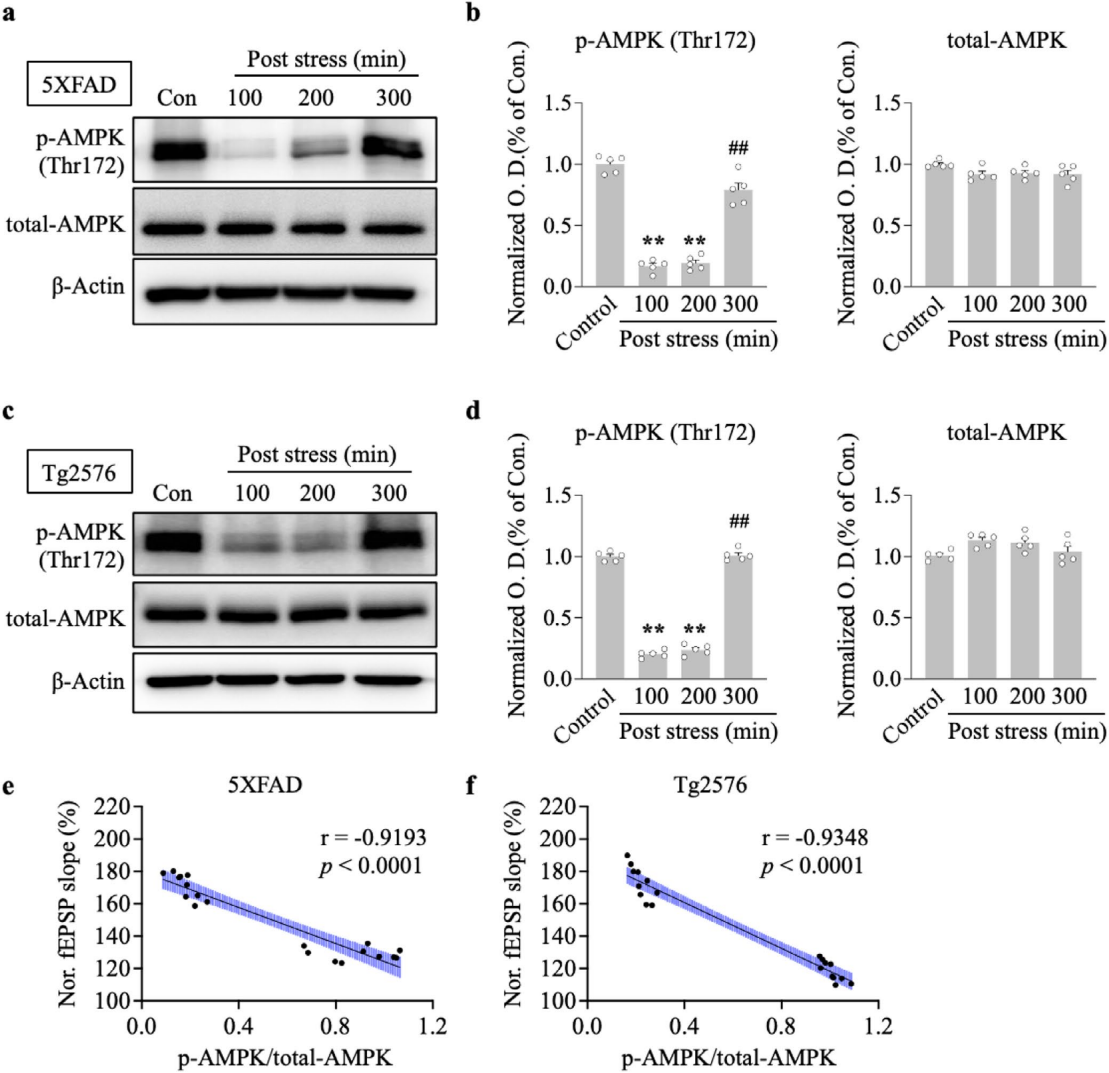
Phospho‐AMPK protein levels were decreased in stressed 5xFAD and Tg2576 mouse hippocampus. Hippocampus were collected at different time point after ARS performed and subjected to western blot analysis. Representative immunoblots showing the amount of p‐AMPK (Thr172) and total‐AMPK in hippocampus from 5XFAD mice (*n* = 5 per group from five animals) (a) and Tg2576 mice (*n* = 5 per group from five animals) (c), β‐Actin as a loading control. (b, d) Densitometry analysis and quantification of western blots. (e, f) Correlation between p‐AMPK/total‐AMPK and normalized fEPSP slope (%) utilized Pearson's correlation analysis. Data are expressed as means ± S.E.M. Statistical analyses were used one‐way ANOVA with Turkey's multiple comparison post hoc test. Differences were considered significant at **p < 0.01 compare to control; ^##^ *p* < 0.01 compare to 200 min post stress. fEPSP, field excitatory postsynaptic potential.

We tested the hypothesis that ARS can alter intrahippocampal ATP levels and downregulate the AMPK activity. We first measured whether 30 min of restraint stress changed the ATP concentration in the hippocampus. Hippocampal slices were analyzed for ATP concentration in stressed and non‐stressed 5xFAD and Tg2576 mice. ARS for 30 min caused an increased ATP concentration in the hippocampus of the stressed group compared to the non‐stressed group for 5xFAD (100 min post stress, ARS: 36.9 ± 3.3 vs. control: 17.4 ± 2.5, *p* < 0.01, *n* = 5, Figure 2a) and Tg2576 mice (100 min post stress, ARS: 41.8 ± 2.3 vs. control: 23.4 ± 1.5, *p* < 0.01, *n* = 5, Figure 2b). Next, we observed AMPK activation by measuring phosphorylated AMPKα1/2 (p‐AMPK) protein levels. Immunostaining results showed that exposure to 30 min restraint stress resulted in robust inhibition of p‐AMPK signals in the hippocampus compared to the non‐stressed group in both 5xFAD (Figure 2c,e) and Tg2576 mice (Figure 2d,f). The inhibition of AMPK activity was accompanied by a decrease in p‐AMPK protein levels at Thr172 in the hippocampus of 5xFAD (100 min vs. control, *p* < 0.01; 200 min vs. control, *p* < 0.01, *n* = 5, Figure 3a,b) and Tg2576 mice (100 min vs. control, *p* < 0.01; 200 min vs. control, *p* < 0.01, *n* = 5, Figure 3c,d). Furthermore, decreased p‐AMPK levels tightly correlated with higher fEPSP slopes, as measured in the hippocampus of 5xFAD (*r* = −0.9193, *p* < 0.0001, Figure 3e) and Tg2576 mice (*r* = −0.9348, *p* < 0.0001, Figure 3f). This indicated that the time course of ARS in LTP maintenance strongly correlated with the time course of AMPK inhibition in 5xFAD mice and Tg2576 mice. These results suggest that ARS alters ATP levels, resulting in the inhibition of AMPK activity, which may play a potential role in hippocampal L‐LTP enhancement.

### 3.3 Acute Restraint Stress Activates the mTOR Pathway

**FIGURE 4:**
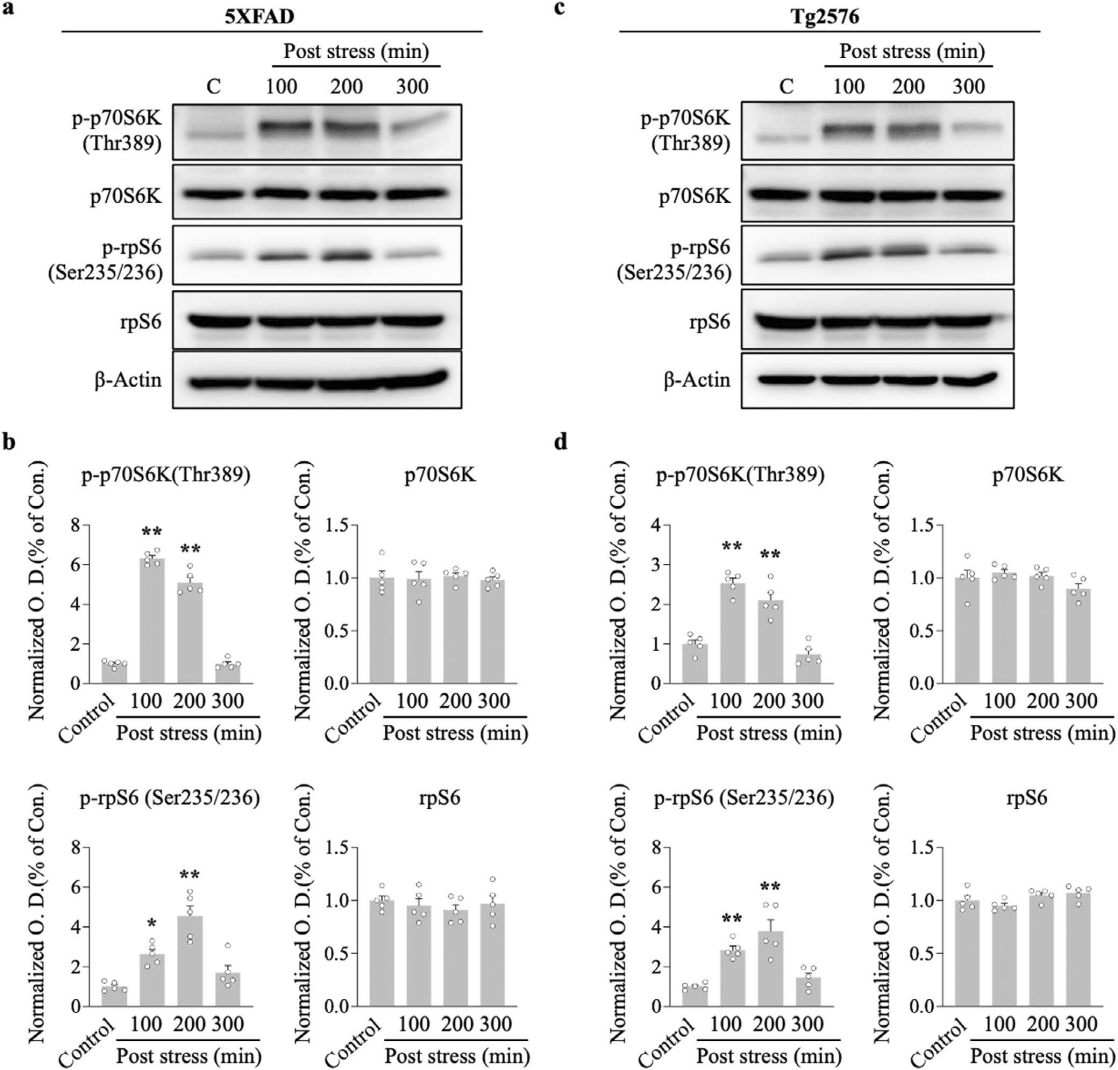
ARS activates the mTOR pathway in hippocampus. Hippocampus were collected at different time point after ARS performed and subjected to western blot analysis. Representative immunoblots showing the amount of p‐p70S6K (Thr389), p70S6K, p‐rpS6 (Ser235/236) and rpS6 in hippocampus from 5XFAD mice (*n* = 5 per group from five animals) (a) and Tg2576 mice (*n* = 5 per group from five animals) (c), β‐Actin as a loading control. (b, d) Densitometry analysis and quantification of western blots. Data are expressed as means ± S.E.M. Statistical analyses were used one‐way ANOVA with Turkey's multiple comparison post hoc test. Differences were considered significant at **p < 0.01 compare to control; *p < 0.05 compare to control.

AMPK is regarded as an upstream mediator of mTOR, and the maintenance of L‐LTP requires protein translation through the mTOR pathway. We conducted western blotting analysis to determine the effect of ARS on the mTOR cascade molecules, p70S6K and its substrate rpS6, because of their established role directly downstream of the mTOR kinase. ARS significantly elevated p‐p70S6K and p‐rpS6 protein levels in the hippocampus compared to the non‐stressed group for 5xFAD (100 min vs. control, *p* < 0.01; 200 min vs. control, *p* < 0.01, *n* = 5, Figure 4a,b) and Tg2576 mice (100 min vs. control, *p* < 0.01; 200 min vs. control, *p* < 0.01, *n* = 5, Figure 4c,d). Notably, the increased effect of ARS on these protein levels persisted for more than 200 min post stress, which correlated with the time course of L‐LTP maintenance in the hippocampi of the two transgenic mice.

### 3.4 Acute Restraint Stress Facilitation of L‐LTP Is Rapamycin Sensitive

**FIGURE 5:**
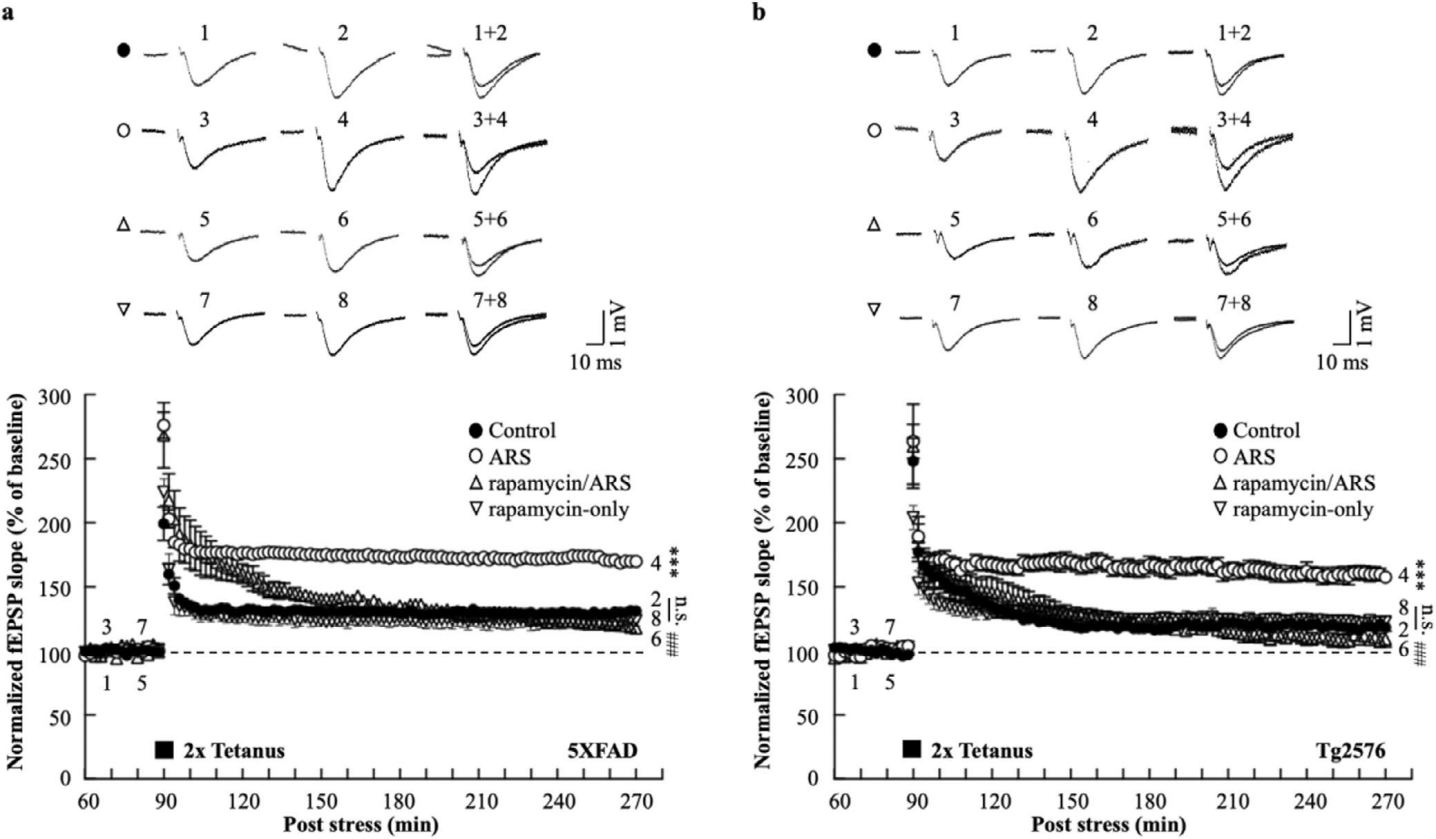
Rapamycin reduces the ARS‐facilitated L‐LTP induction and maintenance. Facilitation of L‐LTP induction (HFS: 2 times tetanic stimulation at 100 Hz, 100 pulses; interstimulus interval, 30s) in the CA1 region of 5xFAD mice (a) and Tg2576 (b) mice hippocampal slices. 1 μM rapamycin prior to ARS (up triangle) inhibits L‐LTP maintenance. No significant difference between the rapamycin‐only group and the control group (down triangle). Control represents non‐stressed AD model mice (closed circle), *n* = 6 per group from six animals. Data are expressed as means ± S.E.M. Differences were considered significant at ***p < 0.001 ARS versus Control, rapamycin vs. Control; ^###^ *p* < 0.001 rapamycin/ARS vs. ARS. Not significant (n.s.) at rapamycin‐only versus Control. fEPSP, Field excitatory postsynaptic potential.

**FIGURE 6:**
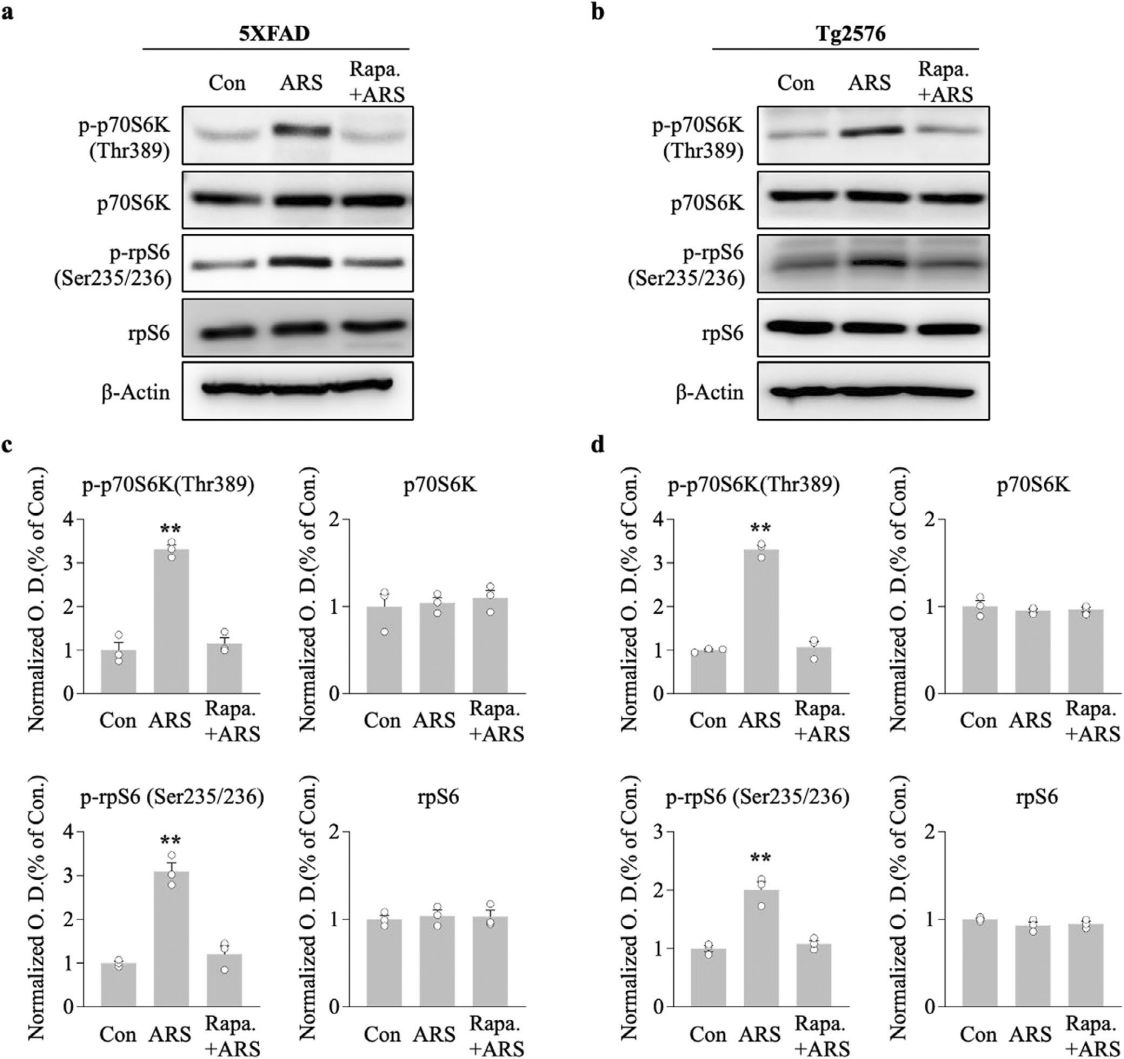
Rapamycin suppresses the ARS‐activated mTOR pathway in hippocampus. (a, b) Representative immunoblot showing the amount of p‐p70S6K (Thr389), p70S6K, p‐rpS6 (Ser235/236) and rpS6 in hippocampus from 5XFAD mice (*n* = 3 per group from three animals) and Tg2576 mice (*n* = 3 per group from three animals), β‐Actin as a loading control. (c, d) Densitometry analysis and quantification of western blots. Data are expressed as means ± S.E.M. Statistical analyses were used one‐way ANOVA with Turkey's multiple comparison post hoc test. Differences were considered significant at **p < 0.01 compare to control. Rapa., Rapamycin.

To explore the direct effect of mTOR on ARS‐facilitated L‐LTP in the hippocampus of AD model mice, we used a mTOR inhibitor, rapamycin (1 μM), prior to the acute stress protocol. The effect of rapamycin on the L‐LTP was assessed using electrophysiological recordings of the hippocampal CA1 region. In hippocampal slices from stressed mice, the fEPSP slope of the L‐LTP was significantly blocked by rapamycin compared to the ARS‐only group for 5xFAD (rapamycin/ARS: 117.8% ± 1.3% vs. ARS: 169.8% ± 3.1%, *p* < 0.001, *n* = 6, Figure 5a) and Tg2576 mice (rapamycin/ARS: 109.2% ± 6.4% vs. ARS: 157.5% ± 4.3%, *p* < 0.001, *n* = 6, Figure 5b). Rapamycin administered prior to the electrophysiological recording did not affect L‐LTP levels, as indicated by a similar fEPSP slope in rapamycin and control slices for 5xFAD (rapamycin‐only: 123.9% ± 4.6% vs. control: 131.2% ± 1.7%, n.s., *n* = 6, Figure 5a) and Tg2576 mice (rapamycin‐only: 122.6% ± 3.7% vs. control: 118.4% ± 2.7%, n.s., *n* = 6, Figure 5b). ARS failed to enhance L‐LTP levels in the presence of rapamycin, suggesting that ARS‐mediated L‐LTP requires the mTOR pathway. To further investigate this, we examined the effect of rapamycin on the ARS‐triggered mTOR pathway in the hippocampi of AD mouse models. We analyzed the phosphorylation of the mTOR cascade components p70S6K and rpS6, and western blotting of hippocampal proteins showed that rapamycin eliminated the ARS‐induced activation of the mTOR pathway in rapamycin‐pretreated mice compared to untreated mice (Figure 6). Taken together, these results suggested that ARS regulates AD mouse hippocampal ATP production and inhibits AMPK activity, which mediates the mTOR pathway involved in the maintenance of L‐LTP.

## 4 Discussion

We first investigated whether 30 min of ARS facilitates a long‐lasting form of synaptic plasticity, termed L‐LTP, in the hippocampal CA1 region of 5xFAD and Tg2576 mice. Second, we highlighted that ARS regulates ATP concentration and significantly inhibits AMPK activity. Importantly, the time course of AMPK phosphorylation strongly correlated with the time course of L‐LTP maintenance by ARS, suggesting a potential connection between AMPK inhibition and L‐LTP maintenance. Third, ARS activated the mTOR pathway through increased phosphorylation of the mTOR components p70S6K and rpS6. Furthermore, rapamycin prevented the enhancement of L‐LTP, suggesting that acute stress inhibits AMPK activity and regulates the mTOR pathway, resulting in the maintenance of L‐LTP in the hippocampus of AD model mice.

Acute stress produces adaptive effects in humans, including enhanced selective attention [38, 39] and improved working memory [40, 41]. Patients with AD exhibit a prominent loss of hippocampus‐dependent memory. Hippocampal synaptic impairment is considered an early alteration in patients with early‐stage AD [42, 43]. One form of prolonged increased synaptic efficacy is LTP, which is widely regarded as the synaptic basis for information storage in the mammalian brain [44]. Many studies have reported a reduction in LTP in AD model mice, such as 5xFAD [45] and Tg2576 mice [46], which develop an age‐dependent motor phenotype in addition to working memory deficits. Therefore, measuring hippocampal synaptic plasticity in AD mouse models serves as a useful indicator of AD‐related synaptic degeneration and memory loss. It has been shown that acute stress or emotional events can facilitate hippocampal LTP [33, 35]. In line with this, our previous study demonstrated that 30 min of ARS rescued impaired LTP in the hippocampus of AD model mice, particularly through glucocorticoid‐mediated effects on hippocampal function, focusing on LTP‐related glutamatergic receptor trafficking and its role in impaired synaptic plasticity [36]. Consistent with these findings, we observed a long‐lasting enhancement of synaptic plasticity in the hippocampal CA1 area in both 5xFAD and Tg2576 mice following ARS. These findings suggest that ARS‐mediated L‐LTP maintenance plays a beneficial role in regulating information storage in AD‐related pathologies. Additionally, given that the effect of ARS appears to be time‐limited in AD‐related pathologies, further studies are needed to develop methods to prolong or optimize this synaptic enhancement, which could have significant clinical applications.

AMPK is an important modulator of cellular energy homeostasis, and its activity is regulated by low neuronal energy [47]. AD has been reported to exert abnormal energy metabolism in neurons [48]. In addition, highly activated AMPK has been observed in AD mice and AD brains [49, 50]. Recently, the AMPK pathway has received increasing attention in neuronal therapeutic research. Several studies have reported that inhibition of AMPK activity has a neuroprotective effect [51, 52]. In the current study, ARS strongly inhibited the phosphorylation of AMPK at Thr172 in the hippocampus of two AD mouse models. These findings highlight the beneficial role of ARS in alleviating AD‐related pathologies by suppressing AMPK activity. AMPK also regulates protein synthesis, which plays an important role in enhancing synaptic plasticity and memory [53]. A previous study reported that pharmacological inhibition of AMPK activity increased L‐LTP maintenance [25]. Consistently, we found that the time course of the AMPK phosphorylation strongly correlated with the time course of L‐LTP maintenance by ARS, suggesting a potential connection between AMPK inhibition and synaptic plasticity improvement under AD conditions.

mTOR downstream substrates are highly expressed in the postsynapse, indicating a local function of mTOR in synaptic plasticity. Studies linking the mTOR pathway to synaptic plasticity were first conducted in invertebrates using rapamycin [54]. Furthermore, L‐LTP requires local protein synthesis, which is disrupted in the rat hippocampus upon exposure to rapamycin [11, 55]. AMPK is a regulator of the mTOR pathway and directly affects L‐LTP maintenance. AMPK activation prevents the L‐LTP expression that is increased by AMPK inhibitors. Moreover, rapamycin has been shown to completely block the effect of an AMPK inhibitor on L‐LTP enhancement [25]. These reports have demonstrated the critical role of the mTOR pathway in regulating synaptic plasticity. In our study, L‐LTP in stressed mice resulted in long‐lasting enhancement of the hippocampal CA1 region. Rapamycin blocked these changes in the maintenance of L‐LTP in the hippocampi of stressed mice.

Previous studies have suggested that physical exercise reduces depressive behaviors, enhances hippocampal‐dependent learning, increases hippocampal neurogenesis, and synaptic plasticity [56, 57]. In fact, the therapeutic effects of physical exercise in treating AD are increasingly recognized [58, 59]. Various mechanisms have been proposed for the beneficial properties of physical exercise in AD, including a reduction in abnormal protein aggregates and neuroinflammation, and a boost in neurogenesis [60]. It was shown that acute stress and exercise share common neural mechanisms [61, 62]. Indicating that appropriately managed acute stress stimulation during daily activities could be beneficial for patients with AD. Moreover, identifying these underlying mechanisms could help promote the development of novel pharmaceutical agents in the treatment of AD. We acknowledge the limitations of the current study, particularly in terms of the behavioral testing used to assess cognitive function. Future studies will explore alternative approaches to extend the duration of the ARS effect, such as repeated physical activity in AD mice, to better accommodate experimental constraints.

## 5 Conclusion

Here, using 5xFAD and Tg2576 mice, we directly measured the hypothesis that acute restraint stress increases hippocampal ATP production, then suppresses the aberrant AMPK activity and removes the inhibition of the mTOR pathway, which rescues the AD‐induced synaptic plasticity impairment (Figure 6). Thus, the acute stress mediating the AMPK‐mTOR pathway is likely to be a beneficial candidate to enhance synaptic functions under AD‐related pathologies.

## Author Contributions

**Ming Wang:** conceptualization, data curation, methodology, investigation, writing – original draft, supervision, funding acquisition. **Baoyuan Jin:** data curation, methodology, investigation, formal analysis, validation, resources, writing – review and editing. **Jihoon Jo:** methodology, supervision, funding acquisition.

## Conflicts of Interest

The authors declare no conflicts of interest.

## Supporting information


Data S1.

## Data Availability

The data that support the findings of this study are available from the corresponding author upon reasonable request.

## References

[1] U. Frey and R. G. Morris , “Synaptic Tagging and Long‐Term Potentiation,” Nature 385 (1997): 533–536. 9020359 10.1038/385533a0

[2] M. A. Lynch , “Long‐Term Potentiation and Memory,” Physiological Reviews 84 (2004): 87–136. 14715912 10.1152/physrev.00014.2003

[3] P. T. Pang and B. Lu , “Regulation of Late‐Phase LTP and Long‐Term Memory in Normal and Aging Hippocampus: Role of Secreted Proteins tPA and BDNF,” Ageing Research Reviews 3 (2004): 407–430. 15541709 10.1016/j.arr.2004.07.002

[4] L. M. Suarez , J. Bustamante , L. M. Orensanz , R. del Martin Rio , and J. M. Solis , “Cooperation of Taurine Uptake and Dopamine D1 Receptor Activation Facilitates the Induction of Protein Synthesis‐Dependent Late LTP,” Neuropharmacology 79 (2014): 101–111. 24225198 10.1016/j.neuropharm.2013.10.035

[5] K. Hara , Y. Maruki , X. Long , et al., “Raptor, a Binding Partner of Target of Rapamycin (TOR), Mediates TOR Action,” Cell 110 (2002): 177–189. 12150926 10.1016/s0092-8674(02)00833-4

[6] M. K. Holz , B. A. Ballif , S. P. Gygi , and J. Blenis , “mTOR and S6K1 Mediate Assembly of the Translation Preinitiation Complex Through Dynamic Protein Interchange and Ordered Phosphorylation Events,” Cell 123 (2005): 569–580. 16286006 10.1016/j.cell.2005.10.024

[7] D. H. Kim , D. D. Sarbassov , S. M. Ali , et al., “mTOR Interacts With Raptor to Form a Nutrient‐Sensitive Complex That Signals to the Cell Growth Machinery,” Cell 110 (2002): 163–175. 12150925 10.1016/s0092-8674(02)00808-5

[8] G. M. Gafford , R. G. Parsons , and F. J. Helmstetter , “Consolidation and Reconsolidation of Contextual Fear Memory Requires Mammalian Target of Rapamycin‐Dependent Translation in the Dorsal Hippocampus,” Neuroscience 182 (2011): 98–104. 21439355 10.1016/j.neuroscience.2011.03.023 PMC3087706

[9] L. Stoica , P. J. Zhu , W. Huang , H. Zhou , S. C. Kozma , and M. Costa‐Mattioli , “Selective Pharmacogenetic Inhibition of Mammalian Target of Rapamycin Complex I (mTORC1) Blocks Long‐Term Synaptic Plasticity and Memory Storage,” Proceedings of the National Academy of Sciences of the United States of America 108 (2011): 3791–3796. 21307309 10.1073/pnas.1014715108 PMC3048125

[10] P. Tsokas , E. A. Grace , P. Chan , et al., “Local Protein Synthesis Mediates a Rapid Increase in Dendritic Elongation Factor 1A After Induction of Late Long‐Term Potentiation,” Journal of Neuroscience 25, no. 24 (2005): 5833–5843, 10.1523/JNEUROSCI.0599-05.2005. 15958750 PMC6724870

[11] S. J. Tang , G. Reis , H. Kang , A. C. Gingras , N. Sonenberg , and E. M. Schuman , “A Rapamycin‐Sensitive Signaling Pathway Contributes to Long‐Term Synaptic Plasticity in the Hippocampus,” Proceedings of the National Academy of Sciences of the United States of America 99 (2002): 467–472. 11756682 10.1073/pnas.012605299 PMC117583

[12] S. M. Goorden , G. M. van Woerden , L. van der Weerd , J. P. Cheadle , and Y. Elgersma , “Cognitive Deficits in Tsc1+/− Mice in the Absence of Cerebral Lesions and Seizures,” Annals of Neurology 62 (2007): 648–655. 18067135 10.1002/ana.21317

[13] R. Muraleedharan and B. Dasgupta , “AMPK in the Brain: Its Roles in Glucose and Neural Metabolism,” FEBS Journal 289 (2022): 2247–2262. 34355526 10.1111/febs.16151

[14] S. Ramamurthy and G. V. Ronnett , “Developing a Head for Energy Sensing: AMP‐Activated Protein Kinase as a Multifunctional Metabolic Sensor in the Brain,” Journal of Physiology 574 (2006): 85–93. 16690704 10.1113/jphysiol.2006.110122 PMC1817796

[15] G. V. Ronnett , S. Ramamurthy , A. M. Kleman , L. E. Landree , and S. Aja , “AMPK in the Brain: Its Roles in Energy Balance and Neuroprotection,” Journal of Neurochemistry 109, no. Suppl 1 (2009): 17–23. 19393004 10.1111/j.1471-4159.2009.05916.x PMC2925428

[16] B. Wang and K. K. Cheng , “Hypothalamic AMPK as a Mediator of Hormonal Regulation of Energy Balance,” International Journal of Molecular Sciences 19, no. 11 (2018): 3552, 10.3390/ijms19113552. 30423881 PMC6274700

[17] G. J. Gowans , S. A. Hawley , F. A. Ross , and D. G. Hardie , “AMP Is a True Physiological Regulator of AMP‐Activated Protein Kinase by Both Allosteric Activation and Enhancing Net Phosphorylation,” Cell Metabolism 18 (2013): 556–566. 24093679 10.1016/j.cmet.2013.08.019 PMC3791399

[18] S. C. Lin and D. G. Hardie , “AMPK: Sensing Glucose as Well as Cellular Energy Status,” Cell Metabolism 27 (2018): 299–313. 29153408 10.1016/j.cmet.2017.10.009

[19] J. S. Oakhill , J. W. Scott , and B. E. Kemp , “AMPK Functions as an Adenylate Charge‐Regulated Protein Kinase,” Trends in Endocrinology and Metabolism 23 (2012): 125–132. 22284532 10.1016/j.tem.2011.12.006

[20] B. De Strooper and E. Karran , “The Cellular Phase of Alzheimer's Disease,” Cell 164 (2016): 603–615. 26871627 10.1016/j.cell.2015.12.056

[21] J. A. Orellana , R. Moraga‐Amaro , R. Ã. DÃaz‐Galarce , et al., “Restraint Stress Increases Hemichannel Activity in Hippocampal Glial Cells and Neurons,” Frontiers in Cellular Neuroscience 9 (2015): 102, 10.3389/fncel.2015.00102. 25883550 PMC4382970

[22] T. Williams , J. Courchet , B. Viollet , J. E. Brenman , and F. Polleux , “AMP‐Activated Protein Kinase (AMPK) Activity Is Not Required for Neuronal Development but Regulates Axogenesis During Metabolic Stress,” Proceedings of the National Academy of Sciences of the United States of America 108 (2011): 5849–5854. 21436046 10.1073/pnas.1013660108 PMC3078367

[23] Y. Z. Ling , Z. Y. Li , H. D. Ou‐Yang , et al., “The Inhibition of Spinal Synaptic Plasticity Mediated by Activation of AMP‐Activated Protein Kinase Signaling Alleviates the Acute Pain Induced by Oxaliplatin,” Experimental Neurology 288 (2017): 85–93. 27856287 10.1016/j.expneurol.2016.11.009

[24] C. Garza‐Lombo , A. Schroder , E. M. Reyes‐Reyes , and R. Franco , “mTOR/AMPK Signaling in the Brain: Cell Metabolism, Proteostasis and Survival,” Current Opinion in Toxicology 8 (2018): 102–110. 30417160 10.1016/j.cotox.2018.05.002 PMC6223325

[25] W. B. Potter , K. J. O'Riordan , D. Barnett , et al., “Metabolic Regulation of Neuronal Plasticity by the Energy Sensor AMPK,” PLoS One 5 (2010): e8996. 20126541 10.1371/journal.pone.0008996 PMC2813866

[26] I. Benilova , E. Karran , and B. De Strooper , “The Toxic Abeta Oligomer and Alzheimer's Disease: An Emperor in Need of Clothes,” Nature Neuroscience 15 (2012): 349–357. 22286176 10.1038/nn.3028

[27] G. M. Shankar , S. Li , T. H. Mehta , et al., “Amyloid‐Beta Protein Dimers Isolated Directly From Alzheimer's Brains Impair Synaptic Plasticity and Memory,” Nature Medicine 14 (2008): 837–842. 10.1038/nm1782 PMC2772133 18568035

[28] D. J. Selkoe and J. Hardy , “The Amyloid Hypothesis of Alzheimer's Disease at 25 Years,” EMBO Molecular Medicine 8 (2016): 595–608. 27025652 10.15252/emmm.201606210 PMC4888851

[29] D. M. Walsh , I. Klyubin , J. V. Fadeeva , et al., “Naturally Secreted Oligomers of Amyloid Beta Protein Potently Inhibit Hippocampal Long‐Term Potentiation In Vivo,” Nature 416 (2002): 535–539. 11932745 10.1038/416535a

[30] L. Schwabe , E. J. Hermans , M. Joels , and B. Roozendaal , “Mechanisms of Memory Under Stress,” Neuron 110 (2022): 1450–1467. 35316661 10.1016/j.neuron.2022.02.020

[31] B. S. McEwen , “Stress and Hippocampal Plasticity,” Annual Review of Neuroscience 22 (1999): 105–122. 10.1146/annurev.neuro.22.1.105 10202533

[32] L. E. B. Bettio , J. S. Thacker , S. P. Rodgers , P. S. Brocardo , B. R. Christie , and J. Gil‐Mohapel , “Interplay Between Hormones and Exercise on Hippocampal Plasticity Across the Lifespan,” Biochimica et Biophysica Acta ‐ Molecular Basis of Disease 1866 (2020): 165821. 32376385 10.1016/j.bbadis.2020.165821

[33] H. Hu , E. Real , K. Takamiya , et al., “Emotion Enhances Learning via Norepinephrine Regulation of AMPA‐Receptor Trafficking,” Cell 131 (2007): 160–173. 17923095 10.1016/j.cell.2007.09.017

[34] I. D. Martijena and V. A. Molina , “The Influence of Stress on Fear Memory Processes,” Brazilian Journal of Medical and Biological Research 45 (2012): 308–313. 22450371 10.1590/S0100-879X2012007500045 PMC3854169

[35] G. Whitehead , J. Jo , E. L. Hogg , et al., “Acute Stress Causes Rapid Synaptic Insertion of Ca2+ −Permeable AMPA Receptors to Facilitate Long‐Term Potentiation in the Hippocampus,” Brain 136 (2013): 3753–3765. 24271563 10.1093/brain/awt293 PMC3859225

[36] M. Wang , V. S. Ramasamy , M. Samidurai , and J. Jo , “Acute Restraint Stress Reverses Impaired LTP in the Hippocampal CA1 Region in Mouse Models of Alzheimer's Disease,” Scientific Reports 9 (2019): 10955. 31358853 10.1038/s41598-019-47452-6 PMC6662902

[37] C. H. Bailey and E. R. Kandel , “Structural Changes Accompanying Memory Storage,” Annual Review of Physiology 55 (1993): 397–426. 10.1146/annurev.ph.55.030193.002145 8466181

[38] R. Hoskin , M. D. Hunter , and P. W. Woodruff , “Stress Improves Selective Attention Towards Emotionally Neutral Left Ear Stimuli,” Acta Psychologica 151 (2014): 214–221. 25086222 10.1016/j.actpsy.2014.06.010

[39] H. Sato , I. Takenaka , and J. I. Kawahara , “The Effects of Acute Stress and Perceptual Load on Distractor Interference,” Quarterly Journal of Experimental Psychology 65 (2012): 617–623. 10.1080/17470218.2011.648944 22463388

[40] M. J. Henckens , E. J. Hermans , Z. Pu , M. Joels , and G. Fernandez , “Stressed Memories: How Acute Stress Affects Memory Formation in Humans,” Journal of Neuroscience 29 (2009): 10111–10119. 19675245 10.1523/JNEUROSCI.1184-09.2009 PMC6664979

[41] E. Y. Yuen , W. Liu , I. N. Karatsoreos , J. Feng , B. S. McEwen , and Z. Yan , “Acute Stress Enhances Glutamatergic Transmission in Prefrontal Cortex and Facilitates Working Memory,” Proceedings of the National Academy of Sciences of the United States of America 106 (2009): 14075–14079. 19666502 10.1073/pnas.0906791106 PMC2729022

[42] S. W. Scheff , D. A. Price , F. A. Schmitt , S. T. DeKosky , and E. J. Mufson , “Synaptic Alterations in CA1 in Mild Alzheimer Disease and Mild Cognitive Impairment,” Neurology 68 (2007): 1501–1508. 17470753 10.1212/01.wnl.0000260698.46517.8f

[43] S. W. Scheff , D. A. Price , F. A. Schmitt , and E. J. Mufson , “Hippocampal Synaptic Loss in Early Alzheimer's Disease and Mild Cognitive Impairment,” Neurobiology of Aging 27 (2006): 1372–1384. 16289476 10.1016/j.neurobiolaging.2005.09.012

[44] R. G. Morris , “Long‐Term Potentiation and Memory,” Philosophical Transactions of the Royal Society of London. Series B, Biological Sciences 358 (2003): 643–647. 12740109 10.1098/rstb.2002.1230 PMC1693171

[45] S. Forner , S. Kawauchi , G. Balderrama‐Gutierrez , et al., “Systematic Phenotyping and Characterization of the 5xFAD Mouse Model of Alzheimer's Disease,” Scientific Data 8 (2021): 270. 34654824 10.1038/s41597-021-01054-y PMC8519958

[46] P. F. Chapman , G. L. White , M. W. Jones , et al., “Impaired Synaptic Plasticity and Learning in Aged Amyloid Precursor Protein Transgenic Mice,” Nature Neuroscience 2 (1999): 271–276. 10195221 10.1038/6374

[47] D. G. Hardie , F. A. Ross , and S. A. Hawley , “AMPK: A Nutrient and Energy Sensor That Maintains Energy Homeostasis,” Nature Reviews. Molecular Cell Biology 13 (2012): 251–262. 22436748 10.1038/nrm3311 PMC5726489

[48] M. T. Lin and M. F. Beal , “Mitochondrial Dysfunction and Oxidative Stress in Neurodegenerative Diseases,” Nature 443 (2006): 787–795. 17051205 10.1038/nature05292

[49] T. Ma , Y. Chen , V. Vingtdeux , et al., “Inhibition of AMP‐Activated Protein Kinase Signaling Alleviates Impairments in Hippocampal Synaptic Plasticity Induced by Amyloid Beta,” Journal of Neuroscience 34 (2014): 12230–12238. 25186765 10.1523/JNEUROSCI.1694-14.2014 PMC4152616

[50] V. Vingtdeux , P. Davies , D. W. Dickson , and P. Marambaud , “AMPK Is Abnormally Activated in Tangle‐ and Pre‐Tangle‐Bearing Neurons in Alzheimer's Disease and Other Tauopathies,” Acta Neuropathologica 121 (2011): 337–349. 20957377 10.1007/s00401-010-0759-x PMC3060560

[51] J. Li , Z. Zeng , B. Viollet , G. V. Ronnett , and L. D. McCullough , “Neuroprotective Effects of Adenosine Monophosphate‐Activated Protein Kinase Inhibition and Gene Deletion in Stroke,” Stroke 38 (2007): 2992–2999. 17901380 10.1161/STROKEAHA.107.490904 PMC2637379

[52] L. D. McCullough , Z. Zeng , H. Li , L. E. Landree , J. McFadden , and G. V. Ronnett , “Pharmacological Inhibition of AMP‐Activated Protein Kinase Provides Neuroprotection in Stroke,” Journal of Biological Chemistry 280 (2005): 20493–20502. 15772080 10.1074/jbc.M409985200

[53] M. Costa‐Mattioli , W. S. Sossin , E. Klann , and N. Sonenberg , “Translational Control of Long‐Lasting Synaptic Plasticity and Memory,” Neuron 61 (2009): 10–26. 19146809 10.1016/j.neuron.2008.10.055 PMC5154738

[54] S. K. Yanow , F. Manseau , J. Hislop , V. F. Castellucci , and W. S. Sossin , “Biochemical Pathways by Which Serotonin Regulates Translation in the Nervous System of Aplysia,” Journal of Neurochemistry 70 (1998): 572–583. 9453551 10.1046/j.1471-4159.1998.70020572.x

[55] S. B. Baltaci , R. Mogulkoc , and A. K. Baltaci , “Molecular Mechanisms of Early and Late LTP,” Neurochemical Research 44 (2019): 281–296. 30523578 10.1007/s11064-018-2695-4

[56] B. D. Eadie , V. A. Redila , and B. R. Christie , “Voluntary Exercise Alters the Cytoarchitecture of the Adult Dentate Gyrus by Increasing Cellular Proliferation, Dendritic Complexity, and Spine Density,” Journal of Comparative Neurology 486 (2005): 39–47. 15834963 10.1002/cne.20493

[57] H. van Praag , B. R. Christie , T. J. Sejnowski , and F. H. Gage , “Running Enhances Neurogenesis, Learning, and Long‐Term Potentiation in Mice,” Proceedings of the National Academy of Sciences of the United States of America 96 (1999): 13427–13431. 10557337 10.1073/pnas.96.23.13427 PMC23964

[58] A. De la Rosa , G. Olaso‐Gonzalez , C. Arc‐Chagnaud , et al., “Physical Exercise in the Prevention and Treatment of Alzheimer's Disease,” Journal of Sport and Health Science 9 (2020): 394–404. 32780691 10.1016/j.jshs.2020.01.004 PMC7498620

[59] N. Zhao , J. Xia , and B. Xu , “Physical Exercise May Exert Its Therapeutic Influence on Alzheimer's Disease Through the Reversal of Mitochondrial Dysfunction via SIRT1‐FOXO1/3‐PINK1‐Parkin‐Mediated Mitophagy,” Journal of Sport and Health Science 10 (2021): 1–3. 32861777 10.1016/j.jshs.2020.08.009 PMC7856556

[60] H. McGurran , J. M. Glenn , E. N. Madero , and N. T. Bott , “Prevention and Treatment of Alzheimer's Disease: Biological Mechanisms of Exercise,” Journal of Alzheimer's Disease 69 (2019): 311–338. 10.3233/JAD-180958 31104021

[61] N. Athanasiou , G. C. Bogdanis , and G. Mastorakos , “Endocrine Responses of the Stress System to Different Types of Exercise,” Reviews in Endocrine & Metabolic Disorders 24 (2023): 251–266. 36242699 10.1007/s11154-022-09758-1 PMC10023776

[62] J. P. Herman , J. M. McKlveen , S. Ghosal , et al., “Regulation of the Hypothalamic‐Pituitary‐Adrenocortical Stress Response,” Comprehensive Physiology 6, no. 2 (2016): 603–621, 10.1002/cphy.c150015. 27065163 PMC4867107

