# Supplementary material for: Acute Restraint Stress Induces Long‐Lasting Synaptic Enhancement by Inhibiting AMPK Activation in AD Model Mice
Source: CNS Neurosci Ther. 2025 Mar 18;31(3):e70335. doi: 10.1111/cns.70335 (PMC11919636; doi:10.1111/cns.70335)

Figure S1. Full unedited blots for Figure 3.

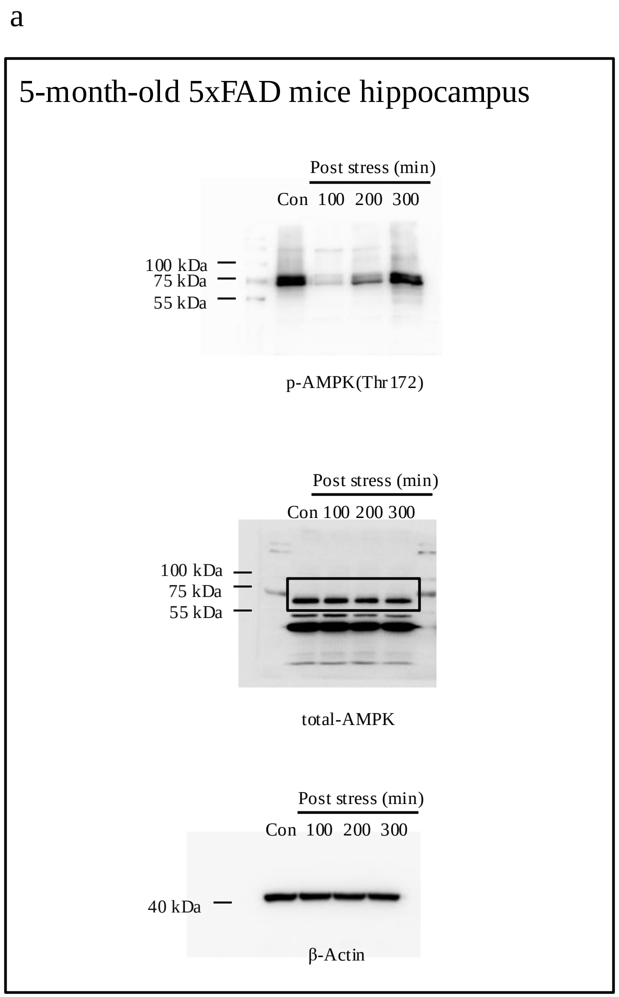

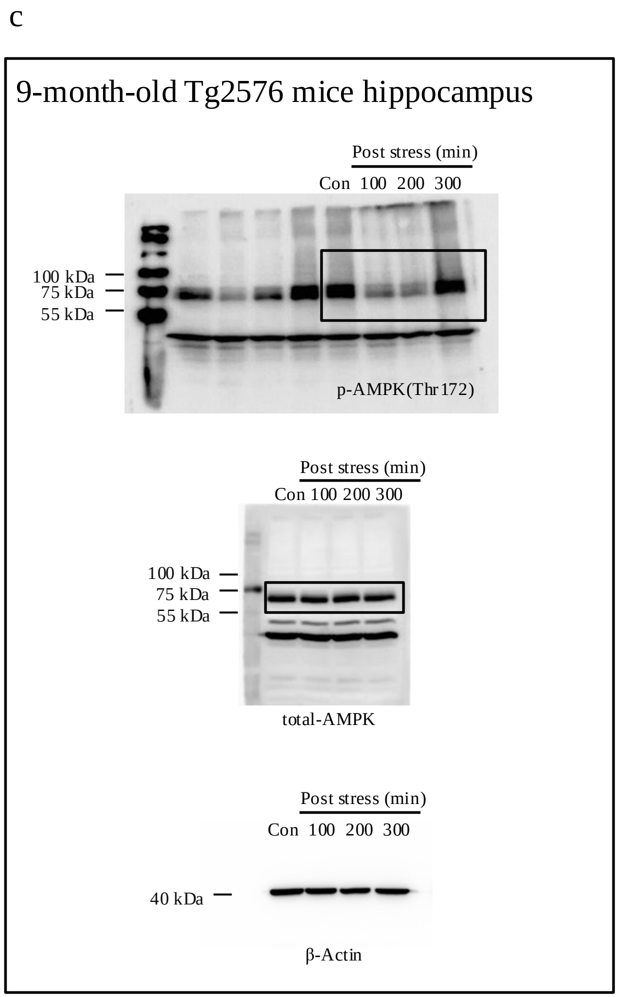

Figure S1. Full unedited blots for Figure 4.

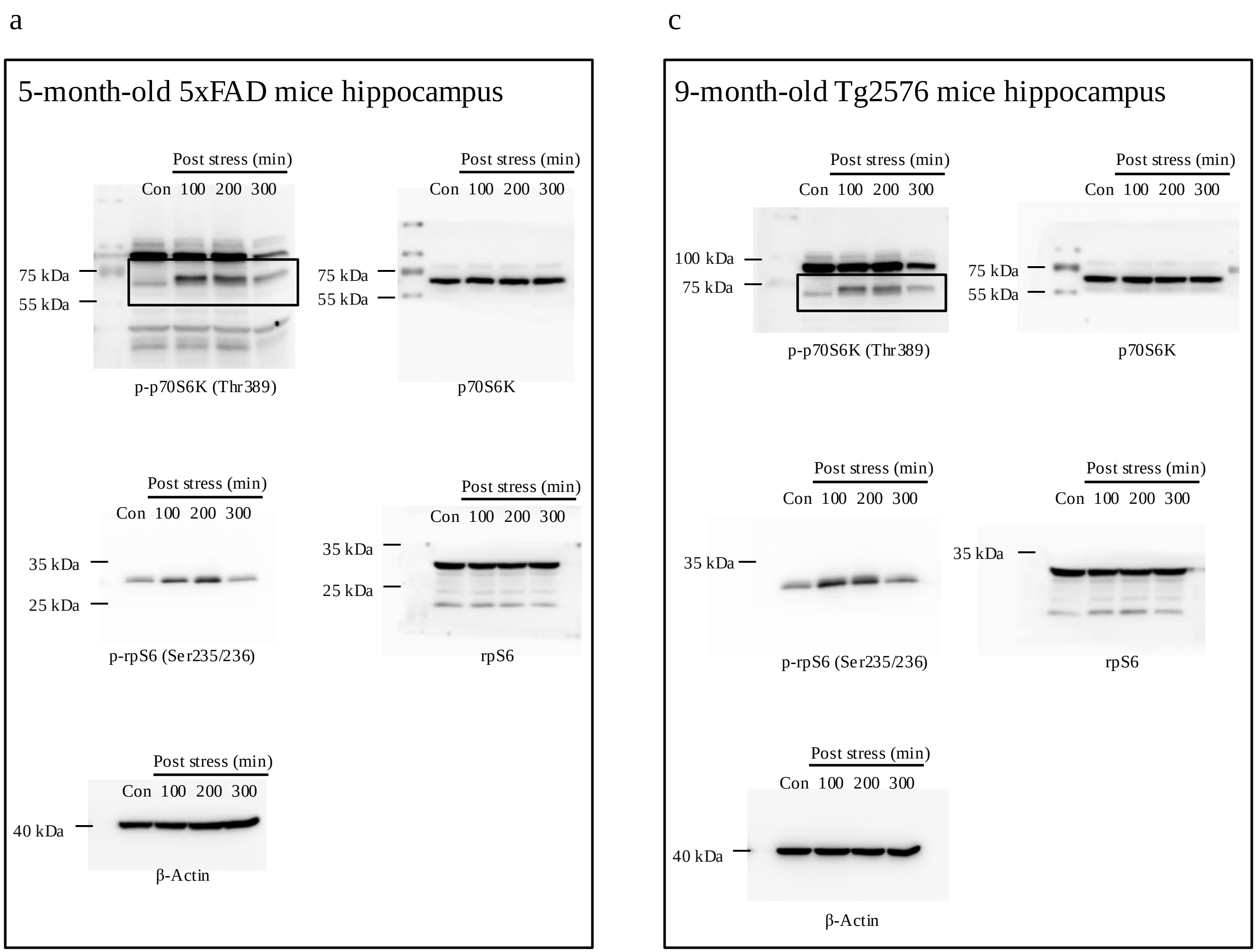

Figure S1. Full unedited blots for Figure 6.

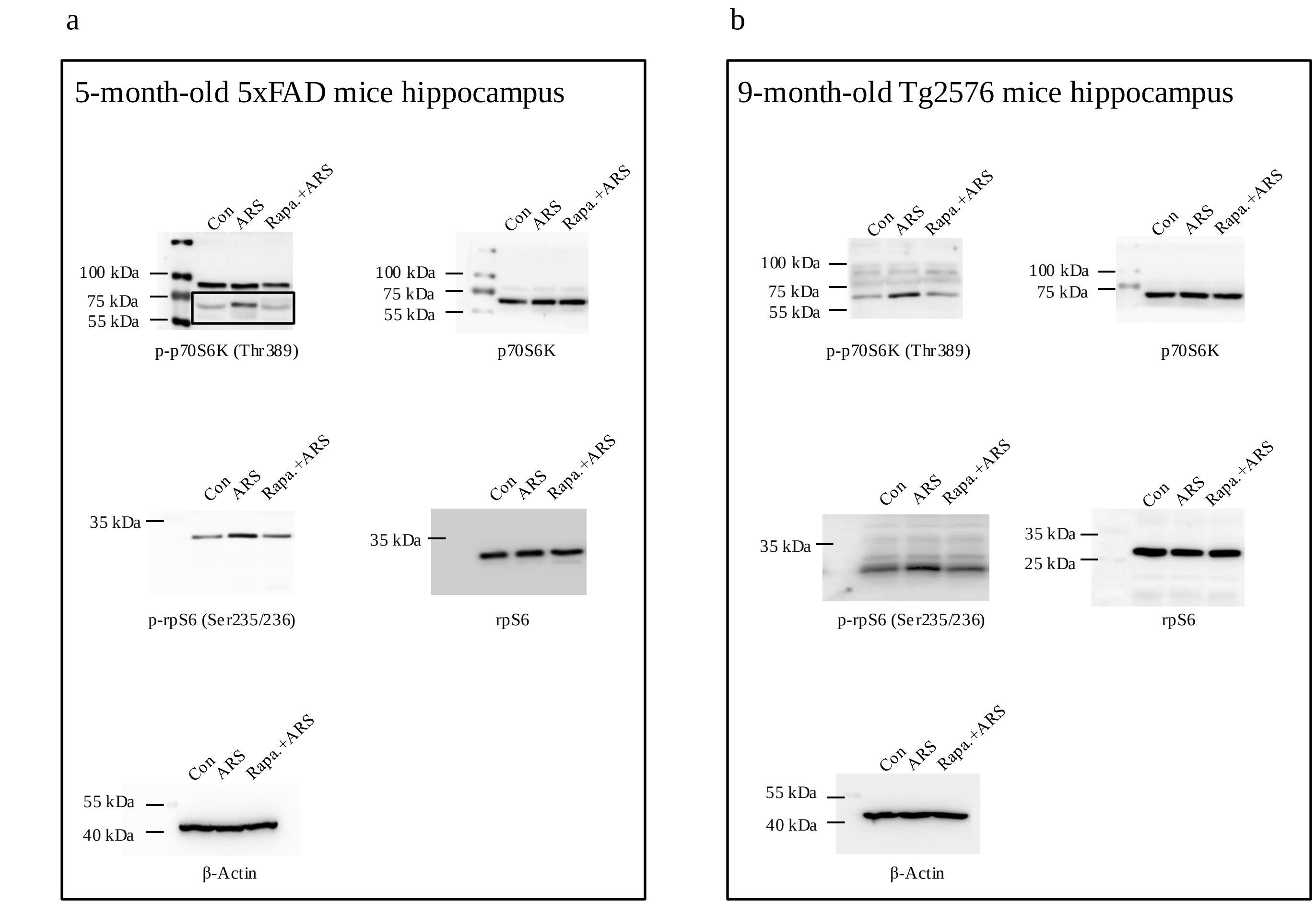

Supplement: Supplementary file 1 — Data S1. [file CNS-31-e70335-s001.pdf]
